# PolED: a manually curated database of functional studies of *POLE* and *POLD1* variants reported in humans

**DOI:** 10.1093/database/baaf076

**Published:** 2025-11-20

**Authors:** Lev Tsarin, Polina V Shcherbakova

**Affiliations:** Eppley Institute for Research in Cancer and Allied Diseases, Fred & Pamela Buffett Cancer Center, University of Nebraska Medical Center, 986805 Nebraska Medical Center, Omaha, NE 68198, United States; Eppley Institute for Research in Cancer and Allied Diseases, Fred & Pamela Buffett Cancer Center, University of Nebraska Medical Center, 986805 Nebraska Medical Center, Omaha, NE 68198, United States

## Abstract

Human *POLE* and *POLD1* genes encode DNA polymerases responsible for genome replication and proofreading of DNA synthesis errors. Germline and somatic *POLE*/*POLD1* mutations compromising the polymerase fidelity cause cancers with high mutational burden. Ultramutation is associated with a better prognosis and immunotherapy response, highlighting the need to define tumour *POLE*/*POLD1* status unambiguously. Prior studies assessed the functional significance of numerous *POLE*/*POLD1* variants in experimental models. However, the data remain scattered and difficult to evaluate by non-specialists, limiting their utility for research and clinical applications. Through manual literature curation, we integrated data from functional studies of clinically relevant *POLE* and *POLD1* variants into PolED, a publicly available database (https://poled-db.org). PolED compiles information on variant effects in biochemical assays, yeast, mammalian cells, and mouse tumour models along with supporting references. It also includes a concise summary of functional significance for each variant. PolED aims to assist in clinical decision-making, guide personalized therapy, and promote further research.

## Introduction

Genome stability relies on multiple mechanisms, including DNA repair pathways, dNTP pools maintenance, accurate DNA replication, and cell cycle checkpoints. Among these, high-fidelity DNA synthesis by replicative polymerases ε (Pol ε) and δ (Pol δ) is the most critical for preventing spontaneous mutations [1, 2]. The catalytic subunits of Pol ε and Pol δ, encoded by the *POLE* and *POLD1* genes in humans, contain a DNA polymerase domain for accurate processive DNA synthesis and an exonuclease domain for correcting rare misincorporation errors. Mutations affecting nucleotide selection by the polymerase domain or proofreading by the exonuclease domain reduce the accuracy of DNA synthesis, increase mutagenesis, and cause cancer predisposition in experimental models [3–17].

Over 12 000 non-silent *POLE/POLD1* variants have been reported in humans (Supplementary Tables S1–S3). A subset of these variants impair the polymerase fidelity and drive the development of tumours with exceptionally high mutation burdens. Somatic *POLE/POLD1* mutations occur across many cancer types [18], but are particularly frequent in colorectal and endometrial cancer [19, 20] and in brain tumours of children with constitutional DNA mismatch repair deficiency (CMMRD) [21, 22]. Germline *POLE/POLD1* mutations cause high-penetrance adult-onset cancer predisposition syndromes [23–26] and have also been associated with paediatric malignancies [27–33]. Ultramutated tumours exhibit improved outcomes due to high neoantigen production and an enhanced anti-tumour immune response. They also respond well to immune checkpoint inhibitors [34, 35]. Thus, *POLE/POLD1* mutation status is a promising predictive biomarker for immunotherapy treatment. Additionally, detecting germline *POLE/POLD1* drivers is crucial for risk assessment and long-term patient management in families with the hereditary syndromes [36, 37].

Distinguishing driver *POLE/POLD1* alleles and benign variants is not straightforward. Pol ε and Pol δ are essential for DNA replication. Mutations result from error-prone DNA synthesis by these enzymes; therefore, only missense variants that preserve robust DNA synthesis capacity could theoretically drive ultramutation. Loss-of-function variants (deletions, insertions, premature stop codons) prevent production of the enzyme and cannot cause ultramutated cancers. The functional impact of most missense variants—except those at catalytic amino acid residues—is difficult to predict without experimental analysis. Since the discovery of Pol ε and Pol δ genes in the late 1980s [38–40], structure-function studies and genetic screens identified many amino acid substitutions that disrupt polymerase fidelity and increase mutation rates [4–7, 10, 12, 13, 15, 16, 41–57]. Some of this information was instrumental in establishing the pathogenicity of first reported human variants. The discovery of a multitude of *POLE*/*POLD1* mutations in human cancers in the 2010s further boosted functional analysis efforts, which now specifically focused on cancer-associated variants with suspected clinical significance [22, 23, 58–85]. Due to the high conservation of polymerase functional regions, yeast model systems have been used extensively to engineer mutations analogous to human variants and determine their impact on the mutation rate. *In vitro* assays with purified Pol ε and Pol δ helped understand the effects of the variants on exonucleolytic proofreading. Some variants have been engineered in cultured human cells and mice to assess the impact on mutation rates, tumour susceptibility, and immunotherapy response. The availability of functional analysis data remains an important consideration in gauging the pathogenicity of new variants. However, much of the available experimental data on *POLE*/*POLD1* variants remains unconsolidated. In the early literature, studies of the polymerase variants do not typically discuss the link to cancer, making these data even more difficult to find. Moreover, interpreting experimental data on mutator phenotypes and DNA polymerase fidelity requires specialized expertise not easily available to clinicians or interdisciplinary researchers.

**Figure 1.**
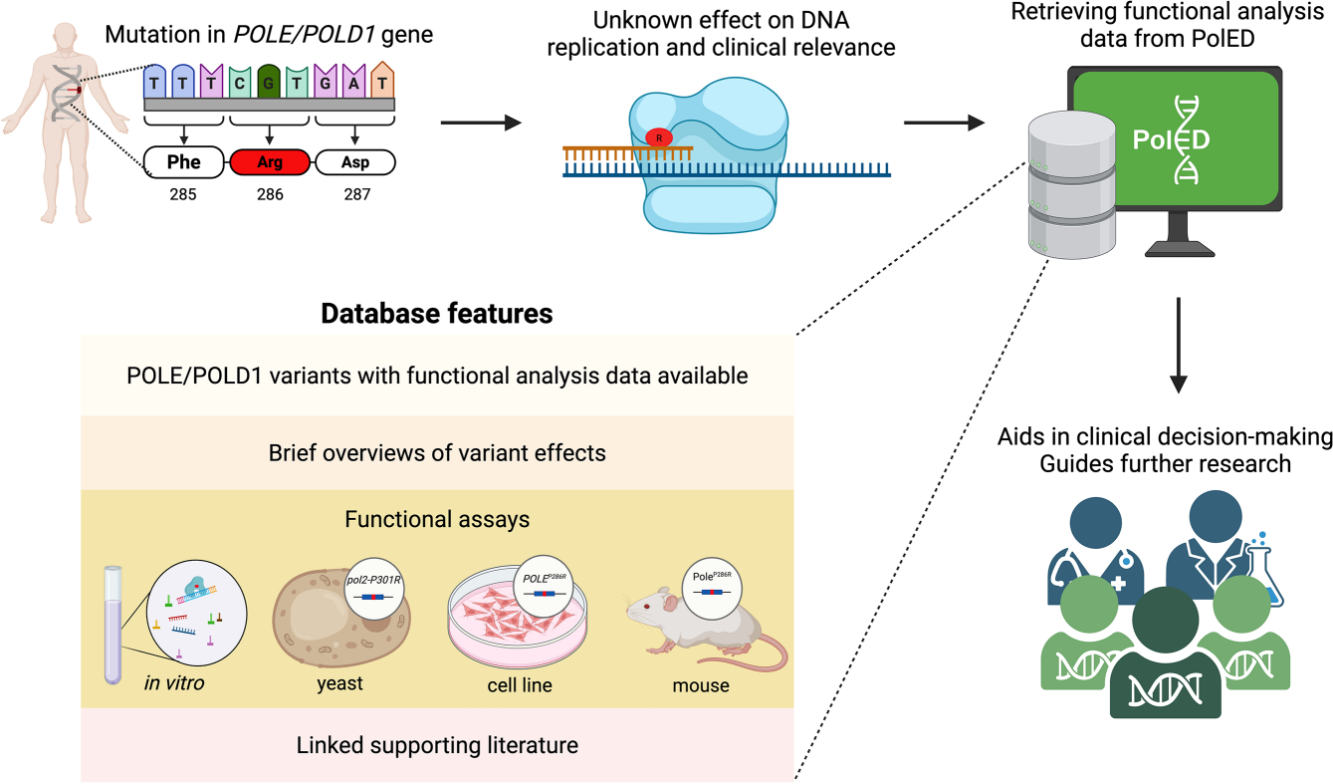
PolED features and application in variant interpretation. Most *POLE*/*POLD1* mutations are classified as variants of unknown significance. PolED compiles functional analysis data on *POLE*/*POLD1* variants reported in humans. This information can assist clinicians and researchers in interpreting clinical variants.

To address these issues, we developed PolED, a manually curated database that compiles functional studies on *POLE* and *POLD1* variants reported in humans. PolED consolidates and structures data from diverse experimental systems—purified proteins, yeast, cultured mammalian cells, and mouse models—to aid in variant classification. The data are reviewed for reliability and presented in a user-friendly format, making them accessible to non-specialists. PolED assists clinicians in making patient management decisions. It also helps researchers navigate through previously investigated variants and design further studies (Fig. 1).

## Data collection and curation

**Figure 2.**
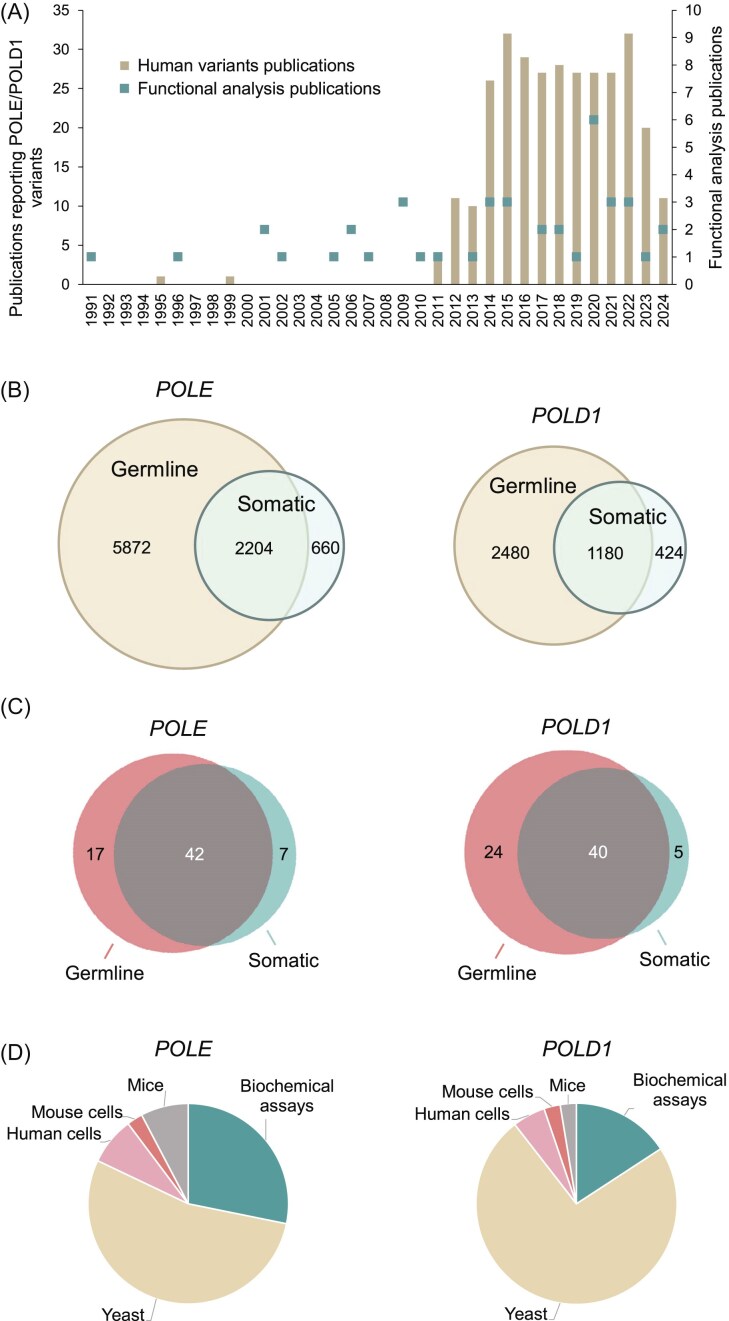
Literature curation and database content. (A) Publications reporting human POLE/POLD1 variants and their functional studies in experimental models. See Supplementary Tables S3 and S4 for the complete list of publications. (B) Venn diagrams of germline and somatic POLE/POLD1 variants reported in the literature and public databases as of August 2024. See Supplementary Tables S1 and S2 for the complete list of variants. (C) Venn diagrams of POLE/POLD1 variants, for which functional analysis data are included in PolED. (D) Summary of PolED data by experimental model.

We assessed 12 820 variants (8736 *POLE* and 4084 *POLD1*; Supplementary Tables S1 and S2) for the availability of functional analysis data. We extracted these variants from cBioPortal, gnomAD, ClinVar, COSMIC, Human Gene Mutation Database (HGMD), The Single Nucleotide Polymorphism Database (dbSNP), Leiden Open Variation Database (LOVD) v.3.0, OncoKB, Cancer Cell Line Encyclopedia (CCLE), and an internal Shcherbakova laboratory literature database maintained since 1990 and cross-checked using PubMed searches using the terms ‘POLE’ and ‘mutation’, or ‘POLD1’ and ‘mutation’ (Fig. 2A; Supplementary Table S3). Most were missense variants, and ∼1.5% were in-frame deletions or insertions. Among the 8736 *POLE* variants, 2204 were reported both in the germline and as somatic mutations in tumours, 5872 only as germline variants, and 660 only as somatic variants. Among the 4084 *POLD1* variants, 1180 were reported in both germline and somatic contexts, 2480 only as germline variants, and 424 only as somatic variants (Fig. 2B).

To collect functional analysis data, we manually curated 90 publications reporting studies of Pol ε and Pol δ variants in experimental assays. We extracted these publications, dating from 1991 to 2025, from the Shcherbakova laboratory literature database cross-checked via extensive PubMed searches. From this literature, we selected data on the variants reported in humans. The data underwent a thorough review for evidence of scientific rigour and statistical significance before inclusion in PolED. At this time, PolED references only original publications reporting a variant’s effect for the first time. Subsequent studies repeating the analysis are cited only if they add new information (e.g. the phenotype of heterozygous *vs*. homozygous cells, biochemical effects on different polymerase subcomplexes, or tissue-specific *vs*. whole-body knock-ins in mice). Figure 2A and Supplementary Table S4 show functional analysis publications included in the current version of PolED. In rare cases, PolED includes unpublished data from our laboratory if there are no analogous published reports. In these cases, the experimental assays use the same methodology and adhere to the same quality standards as our published work. These data are available upon request.

The current PolED version contains functional analysis data for 67 *POLE* and 69 *POLD1* variants. Most occurred both in the germline and as somatic mutations in tumours. Seventeen *POLE* and 24 *POLD1* variants were reported only in the germline, and seven *POLE* and five *POLD1* variants were reported only as somatic mutations (Fig. 2C). Experimental models include *in vitro* biochemical assays with purified polymerases, *in vivo* mutation rate assays in yeast *Saccharomyces cerevisiae* or *Schizosaccharomyces pombe, ex vivo* mutation rate assays in cultured human or mouse cells, and *in vivo* mouse models (Fig. 2D). PolED also provides functional information on 32 catalytic amino acid residue variants with no direct experimental data, because structural and functional studies of other polymerases established the importance of these residues for catalysis of the exonuclease reaction.

## Database design and features

**Figure 3.**
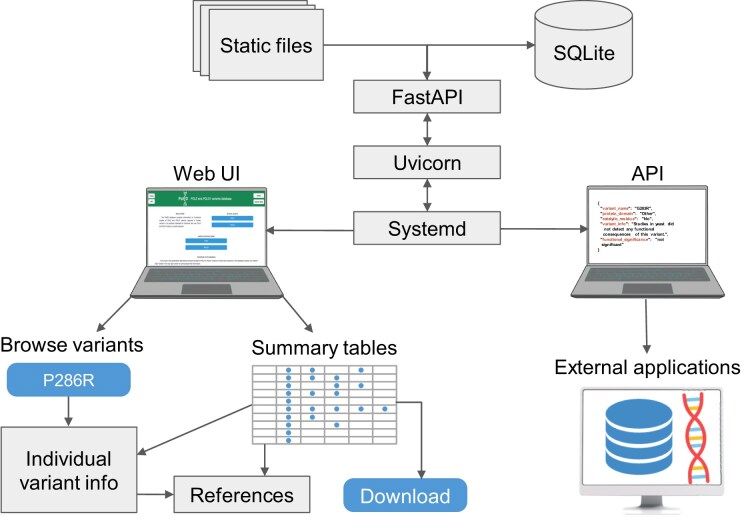
PolED architecture and interface. The PolED database is implemented as a FastAPI-based web application, deployed using Uvicorn, and managed via systemd. The backend stores variant data in an SQLite database and serves both static files and dynamic content. Users can interact with PolED through a web-based interface or programmatically via the API. The interface allows users to explore data on individual variants or view and download summary tables of all variants with demonstrated functional significance. The API enables integration with external tools and workflows (e.g. NGS pipelines, annotation tools, external databases).

The PolED web application is developed using Uvicorn (0.34.1, an ASGI server) and FastAPI (v0.115.12, a Python web framework). It runs in a Python 3.10.12 virtual environment and is managed by systemd on a Linux server (Fig. 3, *top*). Variants and associated information are stored in SQLite (3.37.2) and accessed via SQLAlchemy (2.03.23). The interface is rendered using HTML, CSS, and JavaScript with static content managed by FastAPI. Visual design is powered by Bootstrap (v5.3.5). The interface is adapted for both desktop and mobile browsers. Processing scripts are developed in Python and JavaScript. PolED supports access via modern web browsers, including Chrome, Firefox, and Safari.

Users access the PolED database through an intuitive web interface. It includes options to explore data on individual *POLE*/*POLD1* variants through the variant browser or view and download summary tables of all variants with demonstrated functional significance (Fig. 3, *bottom left*). Variants with evidence of functional significance are marked by colour in the variant browser (*blue* = significant; *grey* = not significant). Each variant has a dedicated page with a brief summary of functional effects and links to detailed summaries of available data from specific experimental systems (biochemical assays, yeast, human cells, mouse cells, or mouse tumour models). Both the variant-specific pages and the summary tables provide hyperlinks to the corresponding literature references. Users can contribute new functional analysis data through the “Submit data” page.

PolED also provides a representational state transfer (REST) application programming interface (API) (Fig. 3, *bottom right*). The API is documented and can be examined in an interactive Swagger UI interface. The API retrieves data in JavaScript Object Notation (JSON) format and supports the following two endpoints:

https://poled-db.org/API/variants/{gene} retrieves all variants for the specified gene, including information about domain location, effect on catalytic residues, a general summary, and functional significance.
https://poled-db.org/API/{variant}/ retrieves available functional analysis data for the specified variant across different experimental models.

## Conclusions and future developments

In this report, we introduce PolED, a web-based database that allows clinicians and researchers to access and analyse functional data on *POLE* and *POLD1* variants in a user-friendly format. By consolidating experimental findings from diverse model systems and presenting them in a structured way, PolED addresses the current gap in the functional annotation of these clinically relevant variants. It emphasizes variant effects on properties relevant to the development of ultramutated cancers: exonuclease and polymerase activities, DNA synthesis fidelity, mutation rate, and tumour susceptibility. The resource facilitates variant interpretation and aids in clinical decision-making, particularly in cancer patient management, where ultramutated phenotypes have emerged as biomarkers for immunotherapy. PolED also helps interpret germline *POLE* and *POLD1* variants to facilitate risk assessment and surveillance of families with cancer predisposition syndromes. Additionally, PolED stimulates further studies of ultramutation in cancer by systematically organizing existing information and providing a catalogue of alleles available as research tools.

We expect that the number of reported *POLE* and *POLD1* variants will grow, and more variants will be examined in experimental assays. We will continue to evaluate new findings and incorporate them into PolED. Data discovered through literature searches or submitted by users are manually curated, processed, and incorporated into the database release within a week of discovery or submission. These data will be of interest to both clinicians and a broad community of researchers in the fields of genome instability and cancer biology.

## Supplementary Material

baaf076_Supplemental_Files

## Data Availability

PolED primarily compiles published data and is freely available at https://poled-db.org/. Unpublished data included in the database are available upon reasonable request to the corresponding author.

## References

[1] Kunkel TA . DNA replication fidelity. J Biol Chem. 2004;279:16895–98. 10.1074/jbc.R400006200 14988392

[2] Kunkel TA . Evolving views of DNA replication (in)fidelity. Cold Spring Harb Symp Quant Biol. 2009;74:91–101. 10.1101/sqb.2009.74.027 19903750 PMC3628614

[3] Albertson TM, Ogawa M, Bugni JM et al. DNA polymerase ε and δ proofreading suppress discrete mutator and cancer phenotypes in mice. Proc Natl Acad Sci USA. 2009;106:17101–4. 10.1073/pnas.0907147106 19805137 PMC2761330

[4] Fazlieva R, Spittle CS, Morrissey D et al. Proofreading exonuclease activity of human DNA polymerase δ and its effects on lesion-bypass DNA synthesis. Nucleic Acids Res. 2009;37:2854–66. 10.1093/nar/gkp155 19282447 PMC2685094

[5] Fortune JM, Stith CM, Kissling GE et al. RPA and PCNA suppress formation of large deletion errors by yeast DNA polymerase δ. Nucleic Acids Res. 2006;34:4335–41. 10.1093/nar/gkl403 16936322 PMC1636344

[6] Ghodgaonkar MM, Kehl P, Ventura I et al. Phenotypic characterization of missense polymerase-δ mutations using an inducible protein-replacement system. Nat Commun. 2014;5:4990. 10.1038/ncomms5990 25241845

[7] Goldsby RE, Hays LE, Chen X et al. High incidence of epithelial cancers in mice deficient for DNA polymerase δ proofreading. Proc Natl Acad Sci USA. 2002;99:15560–5. 10.1073/pnas.232340999 12429860 PMC137756

[8] Goldsby RE, Lawrence NA, Hays LE et al. Defective DNA polymerase-δ proofreading causes cancer susceptibility in mice. Nat Med. 2001;7:638–9. 10.1038/88963 11385474

[9] Korona DA, Lecompte KG, Pursell ZF. The high fidelity and unique error signature of human DNA polymerase ε. Nucleic Acids Res. 2011;39:1763–73. 10.1093/nar/gkq1034 21036870 PMC3061053

[10] Li L, Murphy KM, Kanevets U et al. Sensitivity to phosphonoacetic acid: a new phenotype to probe DNA polymerase δ in *Saccharomyces cerevisiae*. Genetics. 2005;170:569–80. 10.1534/genetics.104.040295 15802517 PMC1450396

[11] Morrison A, Bell JB, Kunkel TA et al. Eukaryotic DNA polymerase amino acid sequence required for 3'-→5' exonuclease activity. Proc Natl Acad Sci USA. 1991;88:9473–7. 10.1073/pnas.88.21.9473 1658784 PMC52740

[12] Pursell ZF, Isoz I, Lundstrom EB et al. Yeast DNA polymerase ε participates in leading-strand DNA replication. Science. 2007;317:127–30. 10.1126/science.1144067 17615360 PMC2233713

[13] Schmitt MW, Matsumoto Y, Loeb LA. High fidelity and lesion bypass capability of human DNA polymerase δ. Biochimie. 2009;91:1163–72. 10.1016/j.biochi.2009.06.007 19540301 PMC2774493

[14] Shcherbakova PV, Pavlov YI, Chilkova O et al. Unique error signature of the four-subunit yeast DNA polymerase ε. J Biol Chem. 2003;278:43770–80. 10.1074/jbc.M306893200 12882968

[15] Simon M, Giot L, Faye G. The 3' to 5' exonuclease activity located in the DNA polymerase δ subunit of *Saccharomyces cerevisiae* is required for accurate replication. EMBO J. 1991;10:2165–70. 10.1002/j.1460-2075.1991.tb07751.x 1648480 PMC452904

[16] Venkatesan RN, Hsu JJ, Lawrence NA et al. Mutator phenotypes caused by substitution at a conserved motif A residue in eukaryotic DNA polymerase δ. J Biol Chem. 2006;281:4486–94. 10.1074/jbc.M510245200 16344551

[17] Venkatesan RN, Treuting PM, Fuller ED et al. Mutation at the polymerase active site of mouse DNA polymerase δ increases genomic instability and accelerates tumorigenesis. Mol Cell Biol. 2007;27:7669–82. 10.1128/MCB.00002-07 17785453 PMC2169052

[18] Cerami E, Gao J, Dogrusoz U et al. The cBio cancer genomics portal: an open platform for exploring multidimensional cancer genomics data. Cancer Discov. 2012;2:401–4. 10.1158/2159-8290.CD-12-0095 22588877 PMC3956037

[19] Cancer Genome Atlas Network. Comprehensive molecular characterization of human colon and rectal cancer. Nature. 2012;487:330–7. 10.1038/nature11252 22810696 PMC3401966

[20] Cancer Genome Atlas Research Network . Integrated genomic characterization of endometrial carcinoma. Nature. 2013;497:67–73. 10.1038/nature12113 23636398 PMC3704730

[21] Erson-Omay EZ, Cağlayan AO, Schultz N et al. Somatic *POLE* mutations cause an ultramutated giant cell high-grade glioma subtype with better prognosis. Neuro Oncol. 2015;17:1356–64. 25740784 10.1093/neuonc/nov027 PMC4578578

[22] Shlien A, Campbell BB, de Borja R et al. Combined hereditary and somatic mutations of replication error repair genes result in rapid onset of ultra-hypermutated cancers. Nat Genet. 2015;47:257–62. 10.1038/ng.3202 25642631

[23] Palles C, Cazier JB, Howarth KM et al. Germline mutations affecting the proofreading domains of POLE and POLD1 predispose to colorectal adenomas and carcinomas. Nat Genet. 2013;45:136–44. 10.1038/ng.2503 23263490 PMC3785128

[24] Rohlin A, Zagoras T, Nilsson S et al. A mutation in *POLE* predisposing to a multi-tumour phenotype. Int J Oncol. 2014;45:77–81. 10.3892/ijo.2014.2410 24788313 PMC4079162

[25] Valle L, Hernandez-Illan E, Bellido F et al. New insights into *POLE* and *POLD1* germline mutations in familial colorectal cancer and polyposis. Hum Mol Genet. 2014;23:3506–12. 10.1093/hmg/ddu058 24501277

[26] Vande Perre P, Siegfried A, Corsini C et al. Germline mutation p.N363K in POLE is associated with an increased risk of colorectal cancer and giant cell glioblastoma. Fam Cancer. 2019;18:173–78. 10.1007/s10689-018-0102-6 30368636

[27] Berrino E, Filippi R, Visintin C et al. Collision of germline *POLE* and *PMS2* variants in a young patient treated with immune checkpoint inhibitors. NPJ Precis Oncol. 2022;6:15. 10.1038/s41698-022-00258-8 35260767 PMC8904527

[28] Lindsay H, Scollon S, Reuther J et al. Germline POLE mutation in a child with hypermutated medulloblastoma and features of constitutional mismatch repair deficiency. Cold Spring Harb Mol Case Stud. 2019;5:a004499. 10.1101/mcs.a004499 31624068 PMC6824253

[29] Michaeli O, Ladany H, Erez A et al. Di-genic inheritance of germline *POLE* and *PMS2* pathogenic variants causes a unique condition associated with pediatric cancer predisposition. Clin Genet. 2022;101:442–47. 10.1111/cge.14106 34967012

[30] Schamschula E, Kinzel M, Wernstedt A et al. Teenage-οnset colorectal cancers in a digenic cancer predisposition syndrome provide clues for the interaction between mismatch repair and polymerase δ proofreading deficiency in tumorigenesis. Biomolecules. 2022;12. 36291559 10.3390/biom12101350 PMC9599501

[31] Sehested A, Meade J, Scheie D et al. Constitutional *POLE* variants causing a phenotype reminiscent of constitutional mismatch repair deficiency. Hum Mutat. 2022;43:85–96. 10.1002/humu.24299 34816535

[32] Tesi B, Lagerstedt-Robinson K, Abel F et al. Diagnostic yield and clinical impact of germline sequencing in children with CNS and extracranial solid tumors—a nationwide, prospective Swedish study. Lancet Reg Health Eur. 2024;39:100881. 10.1016/j.lanepe.2024.100881 38803632 PMC11129334

[33] Wimmer K, Beilken A, Nustede R et al. A novel germline *POLE* mutation causes an early onset cancer prone syndrome mimicking constitutional mismatch repair deficiency. Fam Cancer. 2017;16:67–71. 10.1007/s10689-016-9925-1 27573199 PMC5243902

[34] Kogl J, Pan TL, Marth C et al. The game-changing impact of *POLE* mutations in oncology-a review from a gynecologic oncology perspective. Front Oncol. 2024;14:1369189. 10.3389/fonc.2024.1369189 39239272 PMC11374733

[35] Ma X, Dong L, Liu X et al. *POLE*/*POLD1* mutation and tumor immunotherapy. J Exp Clin Cancer Res. 2022;41:216. 10.1186/s13046-022-02422-1 35780178 PMC9250176

[36] Mur P, Viana-Errasti J, Garcia-Mulero S et al. Recommendations for the classification of germline variants in the exonuclease domain of POLE and POLD1. Genome Med. 2023;15:85. 10.1186/s13073-023-01234-y 37848928 PMC10580551

[37] Palles C, Martin L, Domingo E et al. The clinical features of polymerase proof-reading associated polyposis (PPAP) and recommendations for patient management. Fam Cancer. 2022;21:197–209. 10.1007/s10689-021-00256-y 33948826 PMC8964588

[38] Boulet A, Simon M, Faye G et al. Structure and function of the *Saccharomyces cerevisiae CDC2* gene encoding the large subunit of DNA polymerase III. EMBO J. 1989;8:1849–54. 10.1002/j.1460-2075.1989.tb03580.x 2670563 PMC401032

[39] Morrison A, Araki H, Clark AB et al. A third essential DNA polymerase in *S. cerevisiae*. Cell. 1990;62:1143–51. 10.1016/0092-8674(90)90391-Q 2169349

[40] Sitney KC, Budd ME, Campbell JL. DNA polymerase III, a second essential DNA polymerase, is encoded by the *S. cerevisiae CDC2* gene. Cell. 1989;56:599–605. 10.1016/0092-8674(89)90582-5 2645055

[41] Boyce KJ, Cao C, Xue C et al. A spontaneous mutation in DNA polymerase *POL3* during in vitro passaging causes a hypermutator phenotype in *Cryptococcus* species. DNA Repair (Amst). 2020;86:102751. 10.1016/j.dnarep.2019.102751 31838381 PMC7542539

[42] Darmawan H, Harrison M, Reha-Krantz LJ. DNA polymerase 3'→5' exonuclease activity: different roles of the beta hairpin structure in family-B DNA polymerases. DNA Repair (Amst). 2015;29:36–46. 10.1016/j.dnarep.2015.02.014 25753811

[43] Ganai RA, Bylund GO, Johansson E. Switching between polymerase and exonuclease sites in DNA polymerase ε. Nucleic Acids Res. 2015;43:932–42. 10.1093/nar/gku1353 25550436 PMC4333401

[44] Herr AJ, Ogawa M, Lawrence NA et al. Mutator suppression and escape from replication error-induced extinction in yeast. PLoS Genet. 2011;7:e1002282. 10.1371/journal.pgen.1002282 22022273 PMC3188538

[45] Jin YH, Garg P, Stith CM et al. The multiple biological roles of the 3'→5' exonuclease of *Saccharomyces cerevisiae* DNA polymerase δ require switching between the polymerase and exonuclease domains. Mol Cell Biol. 2005;25:461–71. 10.1128/MCB.25.1.461-471.2005 15601866 PMC538786

[46] Jin YH, Obert R, Burgers PM et al. The 3'→5' exonuclease of DNA polymerase δ can substitute for the 5' flap endonuclease Rad27/Fen1 in processing Okazaki fragments and preventing genome instability. Proc Natl Acad Sci USA. 2001;98:5122–27. 10.1073/pnas.091095198 11309502 PMC33174

[47] Kiktev DA, Dominska M, Zhang T et al. The fidelity of DNA replication, particularly on GC-rich templates, is reduced by defects of the Fe-S cluster in DNA polymerase δ. Nucleic Acids Res. 2021;49:5623–36. 10.1093/nar/gkab371 34019669 PMC8191807

[48] Kirchner JM, Tran H, Resnick MA. A DNA polymerase ε mutant that specifically causes +1 frameshift mutations within homonucleotide runs in yeast. Genetics. 2000;155:1623–32. 10.1093/genetics/155.4.1623 10924461 PMC1461198

[49] Kokoska RJ, Stefanovic L, DeMai J et al. Increased rates of genomic deletions generated by mutations in the yeast gene encoding DNA polymerase δ or by decreases in the cellular levels of DNA polymerase δ. Mol Cell Biol. 2000;20:7490–504. 10.1128/MCB.20.20.7490-7504.2000 11003646 PMC86302

[50] Kokoska RJ, Stefanovic L, Tran HT et al. Destabilization of yeast micro- and minisatellite DNA sequences by mutations affecting a nuclease involved in Okazaki fragment processing (*rad27*) and DNA polymerase δ (*pol3-t*). Mol Cell Biol. 1998;18:2779–88. 10.1128/MCB.18.5.2779 9566897 PMC110657

[51] Murphy K, Darmawan H, Schultz A et al. A method to select for mutator DNA polymerase δs in *Saccharomyces cerevisiae*. Genome. 2006;49:403–10. 10.1139/g05-106 16699561

[52] Nick McElhinny SA, Stith CM, Burgers PM et al. Inefficient proofreading and biased error rates during inaccurate DNA synthesis by a mutant derivative of *Saccharomyces cerevisiae* DNA polymerase δ. J Biol Chem. 2007;282:2324–32. 10.1074/jbc.M609591200 17121822 PMC1839876

[53] Pavlov YI, Shcherbakova PV, Kunkel TA. *In vivo* consequences of putative active site mutations in yeast DNA polymerases α, ε, δ, and ζ. Genetics. 2001;159:47–64. 10.1093/genetics/159.1.47 11560886 PMC1461793

[54] Pursell ZF, Isoz I, Lundstrom EB et al. Regulation of B family DNA polymerase fidelity by a conserved active site residue: characterization of M644W, M644L and M644F mutants of yeast DNA polymerase ε. Nucleic Acids Res. 2007;35:3076–86. 10.1093/nar/gkm132 17452367 PMC1888828

[55] Shcherbakova PV, Noskov VN, Pshenichnov MR et al. Base analog 6-*N*-hydroxylaminopurine mutagenesis in the yeast *Saccharomyces cerevisiae* is controlled by replicative DNA polymerases. Mutat Res. 1996;369:33–44. 10.1016/S0165-1218(96)90045-2 8700180

[56] Stepchenkova EI, Tarakhovskaya ER, Siebler HM et al. Defect of Fe-S cluster binding by DNA polymerase δ in yeast suppresses UV-induced mutagenesis, but enhances DNA polymerase ζ—dependent spontaneous mutagenesis. DNA Repair (Amst). 2017;49:60–9. 10.1016/j.dnarep.2016.11.004 28034630

[57] Williams LN, Herr AJ, Preston BD. Emergence of DNA polymerase ε antimutators that escape error-induced extinction in yeast. Genetics. 2013;193:751–70. 10.1534/genetics.112.146910 23307893 PMC3583996

[58] Barbari SR, Beach AK, Markgren JG et al. Enhanced polymerase activity permits efficient synthesis by cancer-associated DNA polymerase ε variants at low dNTP levels. Nucleic Acids Res. 2022;50:8023–40. 10.1093/nar/gkac602 35822874 PMC9371911

[59] Barbari SR, Kane DP, Moore EA et al. Functional analysis of cancer-associated DNA polymerase ε variants in *Saccharomyces cerevisiae*. G3. 2018;8:1019–29. 10.1534/g3.118.200042 29352080 PMC5844290

[60] Castellsague E, Li R, Aligue R et al. Novel *POLE* pathogenic germline variant in a family with multiple primary tumors results in distinct mutational signatures. Hum Mutat. 2019;40:36–41. 10.1002/humu.23676 30362666

[61] Daee DL, Mertz TM, Shcherbakova PV. A cancer-associated DNA polymerase δ variant modeled in yeast causes a catastrophic increase in genomic instability. Proc Natl Acad Sci USA. 2010;107:157–62. 10.1073/pnas.0907526106 19966286 PMC2806701

[62] Dahl JM, Thomas N, Tracy MA et al. Probing the mechanisms of two exonuclease domain mutators of DNA polymerase ε. Nucleic Acids Res. 2022;50:962–74. 10.1093/nar/gkab1255 35037018 PMC8789060

[63] Demidova EV, Serebriiskii IG, Vlasenkova R et al. Correction: candidate variants in DNA replication and repair genes in early-onset renal cell carcinoma patients referred for germline testing. BMC Genomics. 2023;24:388. 10.1186/s12864-023-09486-z 37095444 PMC10123997

[64] Esteban-Jurado C, Gimenez-Zaragoza D, Munoz J et al. *POLE* and *POLD1* screening in 155 patients with multiple polyps and early-onset colorectal cancer. Oncotarget. 2017;8:26732–43. 10.18632/oncotarget.15810 28423643 PMC5432293

[65] Galati MA, Hodel KP, Gams MS et al. Cancers from novel *Pole*-mutant mouse models provide insights into polymerase-mediated hypermutagenesis and immune checkpoint blockade. Cancer Res. 2020;80:5606–18. 10.1158/0008-5472.CAN-20-0624 32938641 PMC8218238

[66] Hamzaoui N, Alarcon F, Leulliot N et al. Genetic, structural, and functional characterization of *POLE* polymerase proofreading variants allows cancer risk prediction. Genet Med. 2020;22:1533–41. 10.1038/s41436-020-0828-z 32424176

[67] Herzog M, Alonso-Perez E, Salguero I et al. Mutagenic mechanisms of cancer-associated DNA polymerase ε alleles. Nucleic Acids Res. 2021;49:3919–31. 10.1093/nar/gkab160 33764464 PMC8053093

[68] Hodel KP, Sun MJS, Ungerleider N et al. POLE mutation spectra are shaped by the mutant allele identity, Its abundance, and mismatch repair status. Mol Cell. 2020;78:1166–77e1166. 10.1016/j.molcel.2020.05.012 32497495 PMC8177757

[69] Kane DP, Shcherbakova PV. A common cancer-associated DNA polymerase ε mutation causes an exceptionally strong mutator phenotype, indicating fidelity defects distinct from loss of proofreading. Cancer Res. 2014;74:1895–901. 10.1158/0008-5472.CAN-13-2892 24525744 PMC4310866

[70] Labrousse G, Vande Perre P, Parra G et al. The hereditary N363K POLE exonuclease mutant extends PPAP tumor spectrum to glioblastomas by causing DNA damage and aneuploidy in addition to increased mismatch mutagenicity. NAR Cancer. 2023;5:zcad011. 36915289 10.1093/narcan/zcad011 PMC10006997

[71] Lee M, Eng G, Barbari SR et al. Homologous recombination repair truncations predict hypermutation in microsatellite stable colorectal and endometrial tumors. Clin Transl Gastroenterol. 2020;11:e00149. 10.14309/ctg.0000000000000149 32352724 PMC7145036

[72] Li HD, Cuevas I, Zhang M et al. Polymerase-mediated ultramutagenesis in mice produces diverse cancers with high mutational load. J Clin Invest. 2018;128:4179–91. 10.1172/JCI122095 30124468 PMC6118636

[73] Li HD, Lu C, Zhang H et al. A *Pole^P286R^* mouse model of endometrial cancer recapitulates high mutational burden and immunotherapy response. JCI Insight. 2020;5:e138829. 10.1172/jci.insight.138829 32699191 PMC7453891

[74] Liu J, Liu Y, Fu J et al. Preliminary study on the function of the *POLD1* (*CDC2*) EXON2 c.56G>A mutation. Mol Genet Genomic Med. 2020;8:e1280. 32432416 10.1002/mgg3.1280 PMC7434749

[75] Mertz TM, Baranovskiy AG, Wang J et al. Nucleotide selectivity defect and mutator phenotype conferred by a colon cancer-associated DNA polymerase δ mutation in human cells. Oncogene. 2017;36:4427–33. 10.1038/onc.2017.22 28368425 PMC5542868

[76] Mertz TM, Sharma S, Chabes A et al. Colon cancer-associated mutator DNA polymerase δ variant causes expansion of dNTP pools increasing its own infidelity. Proc Natl Acad Sci USA. 2015;112:E2467–76. 10.1073/pnas.1422934112 25827231 PMC4434702

[77] Mur P, Garcia-Mulero S, Del Valle J et al. Role of *POLE* and *POLD1* in familial cancer. Genet Med. 2020;22:2089–100. 10.1038/s41436-020-0922-2 32792570 PMC7708298

[78] Ostroverkhova D, Tyryshkin K, Beach AK et al. DNA polymerase ε and δ variants drive mutagenesis in polypurine tracts in human tumors. Cell Rep. 2024;43:113655. 10.1016/j.celrep.2023.113655 38219146 PMC10830898

[79] Pai CC, Heitzer E, Bertrand S et al. Using canavanine resistance to measure mutation rates in *Schizosaccharomyces pombe*. PLoS One. 2023;18:e0271016. 10.1371/journal.pone.0271016 36626373 PMC9831302

[80] Rocque MJ, Leipart V, Kumar Singh A et al. Characterization of *POLE* c.1373A >T p.(Tyr458Phe), causing high cancer risk. Mol Genet Genomics. 2023;298:555–66. 36856825 10.1007/s00438-023-02000-w PMC10133059

[81] Shinbrot E, Henninger EE, Weinhold N et al. Exonuclease mutations in DNA polymerase epsilon reveal replication strand specific mutation patterns and human origins of replication. Genome Res. 2014;24:1740–50. 10.1101/gr.174789.114 25228659 PMC4216916

[82] Shioi S, Shimamoto A, Song Y et al. DNA polymerase delta Exo domain stabilizes mononucleotide microsatellites in human cells. DNA Repair (Amst). 2021;108:103216. 10.1016/j.dnarep.2021.103216 34530183

[83] Soriano I, Vazquez E, De Leon N et al. Expression of the cancer-associated DNA polymerase ε P286R in fission yeast leads to translesion synthesis polymerase dependent hypermutation and defective DNA replication. PLoS Genet. 2021;17:e1009526. 10.1371/journal.pgen.1009526 34228709 PMC8284607

[84] Weber CAM, Kronke N, Volk V et al. Rare germline variants in *POLE* and *POLD1* encoding the catalytic subunits of DNA polymerases ε and δ in glioma families. Acta Neuropathol Commun. 2023;11:184. 10.1186/s40478-023-01689-5 37990341 PMC10664377

[85] Xing X, Kane DP, Bulock CR et al. A recurrent cancer-associated substitution in DNA polymerase ε produces a hyperactive enzyme. Nat Commun. 2019;10:374. 10.1038/s41467-018-08145-2 30670691 PMC6343027

